# Identification of Genomic Features in Environmentally Induced Epigenetic Transgenerational Inherited Sperm Epimutations

**DOI:** 10.1371/journal.pone.0100194

**Published:** 2014-06-17

**Authors:** Carlos Guerrero-Bosagna, Shelby Weeks, Michael K. Skinner

**Affiliations:** 1 Center for Reproductive Biology, School of Biological Sciences, Washington State University, Pullman, Washington, United States of America; 2 Department of Physics, Biology and Chemistry, Linköping University, Linköping, Sweden; University of Hawaii at Manoa, John A. Burns School of Medicine, United States of America

## Abstract

A variety of environmental toxicants have been shown to induce the epigenetic transgenerational inheritance of disease and phenotypic variation. The process involves exposure of a gestating female and the developing fetus to environmental factors that promote permanent alterations in the epigenetic programming of the germline. The molecular aspects of the phenomenon involve epigenetic modifications (epimutations) in the germline (e.g. sperm) that are transmitted to subsequent generations. The current study integrates previously described experimental epigenomic transgenerational data and web-based bioinformatic analyses to identify genomic features associated with these transgenerationally transmitted epimutations. A previously identified genomic feature associated with these epimutations is a low CpG density (<12/100bp). The current observations suggest the transgenerational differential DNA methylation regions (DMR) in sperm contain unique consensus DNA sequence motifs, zinc finger motifs and G-quadruplex sequences. Interaction of molecular factors with these sequences could alter chromatin structure and accessibility of proteins with DNA methyltransferases to alter de novo DNA methylation patterns. G-quadruplex regions can promote the opening of the chromatin that may influence the action of DNA methyltransferases, or factors interacting with them, for the establishment of epigenetic marks. Zinc finger binding factors can also promote this chromatin remodeling and influence the expression of non-coding RNA. The current study identified genomic features associated with sperm epimutations that may explain in part how these sites become susceptible for transgenerational programming.

## Introduction

A number of environmental factors have been shown to induce the epigenetic transgenerational inheritance of disease and phenotypic variation [1], [2], [3], [4], [5], [6]. The initiation of this transgenerational inheritance process involves exposure of a gestating female and the developing fetus during gonadal sex determination to environmental factors (e.g. toxicants). The exposures promote alterations in the epigenetic programming of the germline that are transmitted to subsequent generations [3], [6], [7]. A variety of environmental toxicants have been shown to induce the epigenetic transgenerational inheritance of disease including the fungicide vinclozolin [1], [3], [4], dioxin [2], [6], pesticides [5], [6], jet fuel hydrocarbons [8] and platicizers (i.e. bisphenol A (BPA) and phthalates) [6]. Environmentally-induced epigenetic modifications in the germline have been shown to involve DNA methylation changes that are transmitted transgenerationally [6]. These germline epigenetic modifications also induce epigenetic alterations in somatic tissues which correlate with transgenerational transcriptome changes [9] and phenotypic abnormalities [10].

Germline epigenetic transgenerational inheritance has been described in several different organisms including plants, flies, worms, rodents, and humans [3], [6], [11], [12], [13], [14], [15]. The role of the germline in the transgenerational process is crucial since it is the only cell that transmits genetic material and stable epigenetic marks (e.g. imprinted genes) to subsequent generations. The initiation of germline development involves a major epigenetic reprogramming through alterations in DNA methylation [16], [17], [18]. DNA methylation erasure takes place during the migration of primordial germ cells to the genital ridge (before colonization of the gonads), while re-methylation is initiated during gonadal sex determination in a sex specific manner [19], [20]. This reprogramming of DNA methylation and the occurrence of other major epigenetic events during primordial germ cell development [21] represents a critical window of exposure for environmental factors [22]. Environmental exposures [23], [24] and epigenetic alterations [25] in this developmental window have been shown to promote the epigenetic transgenerational inheritance of disease and phenotypic variation.

Previous studies have shown that different exposures produce distinct sets of transgenerationally altered differential DNA methylation regions (DMR) in male germ cells, termed epimutations [6]. Interestingly, the transgenerationally altered sperm epimutations among these different exposure groups were found to have minimal overlap [6]. The methylation status of these DMR appears to be transmitted transgenerationally in similar ways to DNA methylation transmission of imprinted genes (imprinted-like mechanism). The DMR identified in these previous studies were found to be exposure specific suggesting potential genomic features among these distinct DMR may exist. The current study was designed to elucidate the potential molecular mechanisms involved in the susceptibility of these epimutations to escape the DNA methylation erasure following fertilization and become transgenerationally programmed.

Recent studies have investigated a variety of DNA-protein interactions that are involved in the establishment of DNA methylation. For example, the presence of protein binding factors in CpG-poor regulatory regions is one feature that would influence DNA methylation [26]. The configuration of specific protein/DNA complexes, formed for example by CTCF or Sp1, can prevent local actions of *de novo* methyltransferases in the genome [27]. The presence of repeat elements has also been reported to influence the establishment of DNA methylation. For example, the presence of CTG/CAG repeats act as a DNA methylation sensitive insulator [28]. Features such as repeat element composition in genomic domains [29] or the composition of nucleotides flanking CpG sites [30] are shown to influence the susceptibility of methylation by Dnmt3 methytransferases. Chromatin marks of histone binding are also shown to correlate with genomic domains in which DNA methylation changes occur [31], [32]. The current study used a bioinformatic analysis of patterns of DNA sequence (motifs) in previously identified exposure specific sets of transgenerational DMR. Observations provide insights into the DNA sequence motifs potentially involved in the establishment of these transgenerational sperm epimutations.

A variety of approaches have been used to identify DNA sequence patterns (motifs) that could be functionally relevant from the perspective of gene expression regulation [33], [34]. These DNA motifs are known to serve as binding sites for transcription factors and other regulatory factors [33]. Methods to identify DNA motifs have evolved from the visual alignment of a few sequences to the use of complex algorithms and computer programs [34]. Identification of consensus sequences or position weight matrices in genomic regions characterize these DNA motifs [34]. Recently, several algorithms have been developed to identify DNA motifs in a given set of sequences and to determine if they are over-represented compared to that expected by chance [33]. The integration of these computational analyses with experimental techniques is becoming fundamental to identify genome-scale regulatory elements [35], [36], [37]. Examples of recent studies using motif analysis at a genomic scale include genome-wide identification of estrogen receptor binding sites [38], identification of CTCF-binding sites in the human genome [39] and identification of motifs associated with aberrant CpG island methylation [40]. The current study integrates previously described experimental epigenomic transgenerational data and web-based bioinformatic analyses to identify DNA motifs to help elucidate the molecular mechanisms involved in environmentally induced transgenerational inheritance of sperm epimutations.

## Results

**Figure 1:**
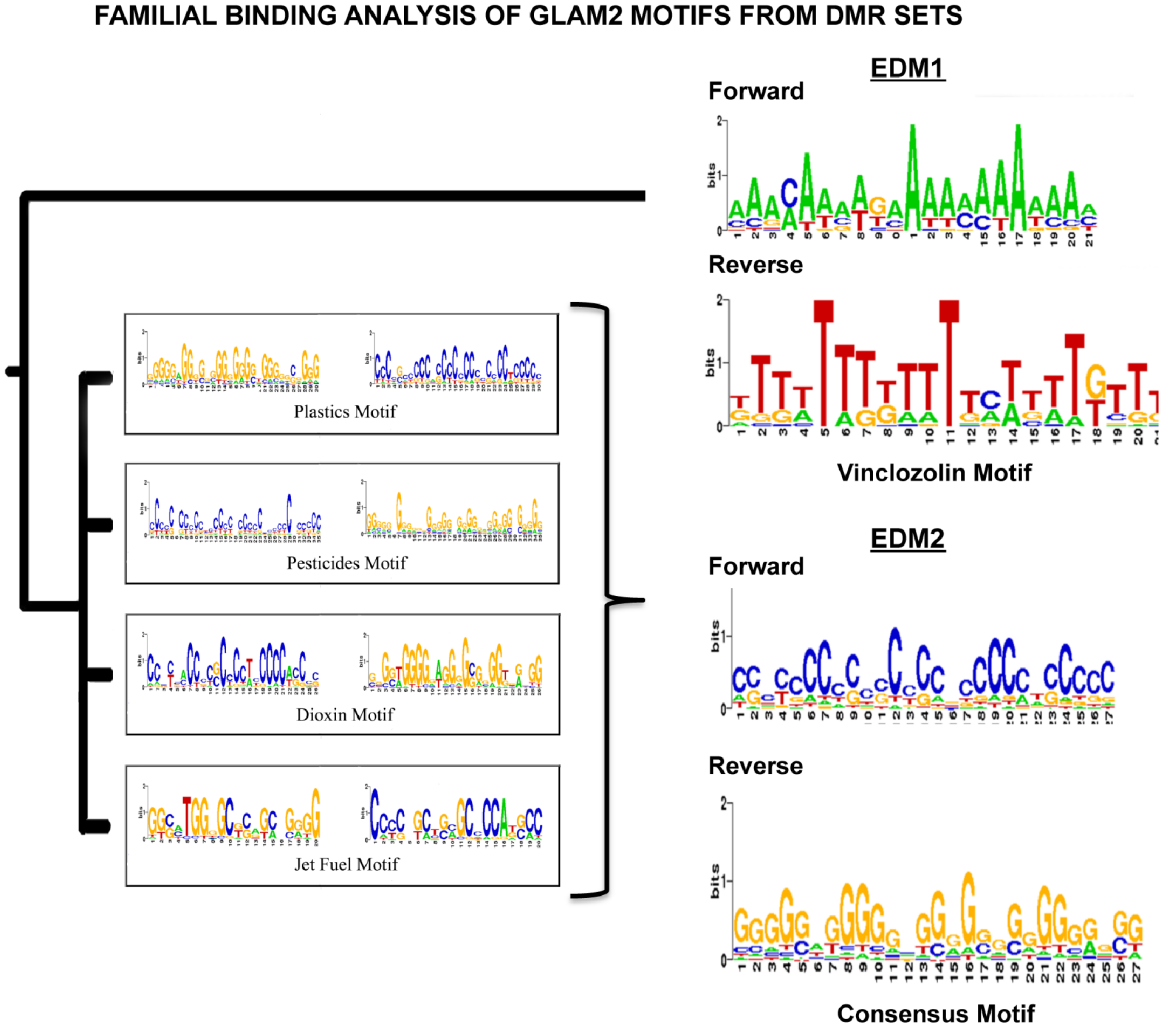
Exposure specific DMR set DNA sequence motifs using GLAM2 and the familial binding tree. The forward (left) and reverse (right) sequences for each motif are presented. The consensus EDM2 motif is presented.

The main goal of the current study was to identify genomic features associated with the environmentally induced epigenetic transgenerational inherited sperm epimutations. Previously described transgenerational differential DNA methylation regions (DMR) in the rat sperm were investigated [3], [6]. These DMR were identified using a methylated DNA immunoprecipitation (MeDIP) followed by genome wide promoter tiling array (Chip) for an MeDIP-Chip protocol previously described [3], [6]. The web-based bioinformatics tool GLAM2 (Gapped Local Alignment of Motifs) [41], which is part of MEME suite [42], was used to identify DNA motifs associated with these transgenerational sperm epimutations. DNA motifs were built from different sets of germline transgenerational DMR derived from different environmental exposures and compared. These were then grouped based on similarities using a familial binding analysis available in the web-based tool for motif analysis termed STAMP [43]. The association tree produced showed two groups of motifs. One branch of the tree was represented only by the “environmentally induced DNA methylation motif 1″ (EDM1) (vinclozolin) previously identified [3], while another branch included motifs from the other exposures (plastics, pesticides, dioxin and jet fuel [6]). A familial binding motif representing this branch was named “environmentally induced DNA methylation motif 2″ (EDM2) (Figure 1). Interestingly, EDM1 is A/T rich while EDM2 is G rich.

**Figure 2:**
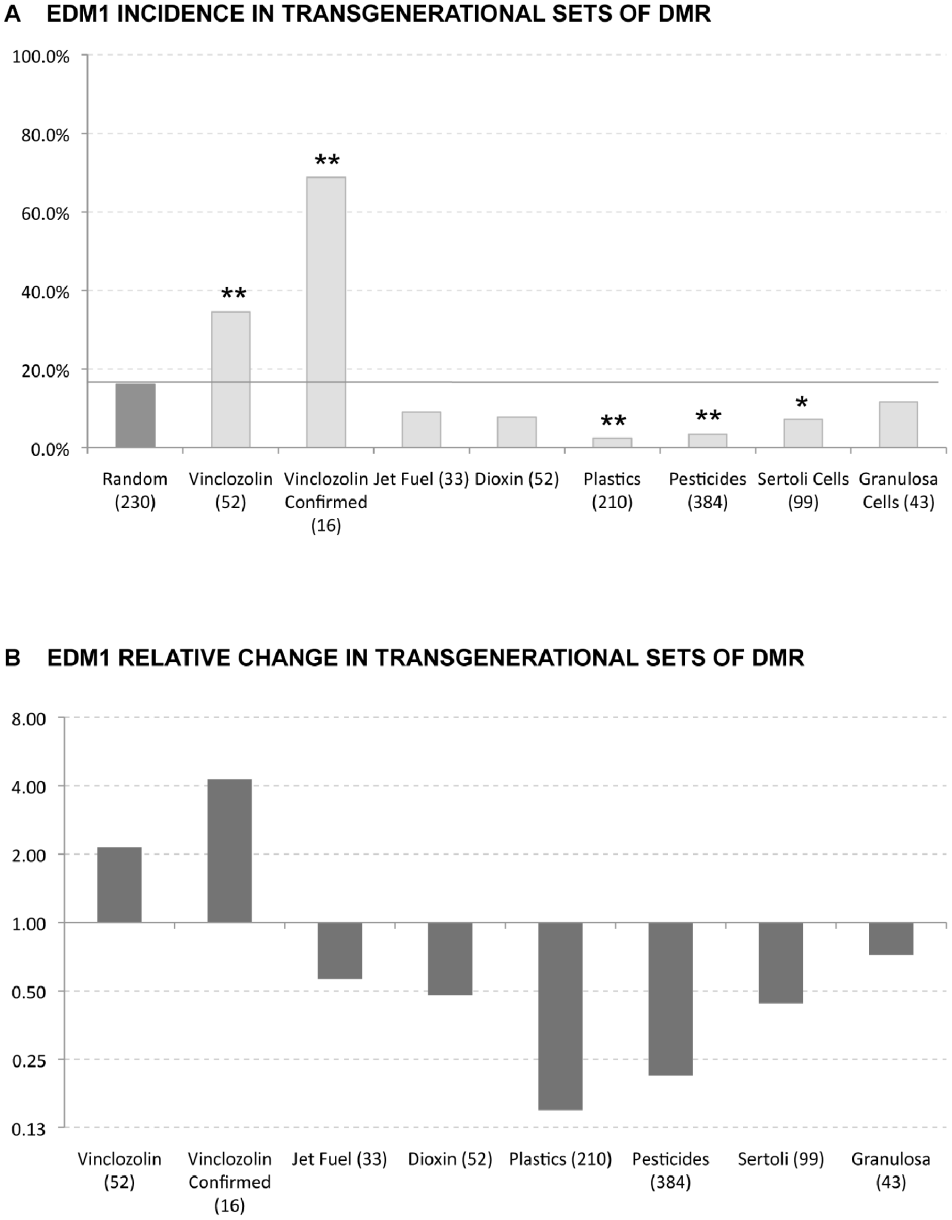
EDM1 incidence in exposure specific epimutation data sets. (A) Individual occurrence (percentage) of EDM1 in a variety of sets with transgenerational DMR and (B) relative change of EDM1 in these DMR. Columns with ** represent significant change with p<0.01, while columns with * represent significant change with p<0.05.

**Figure 3:**
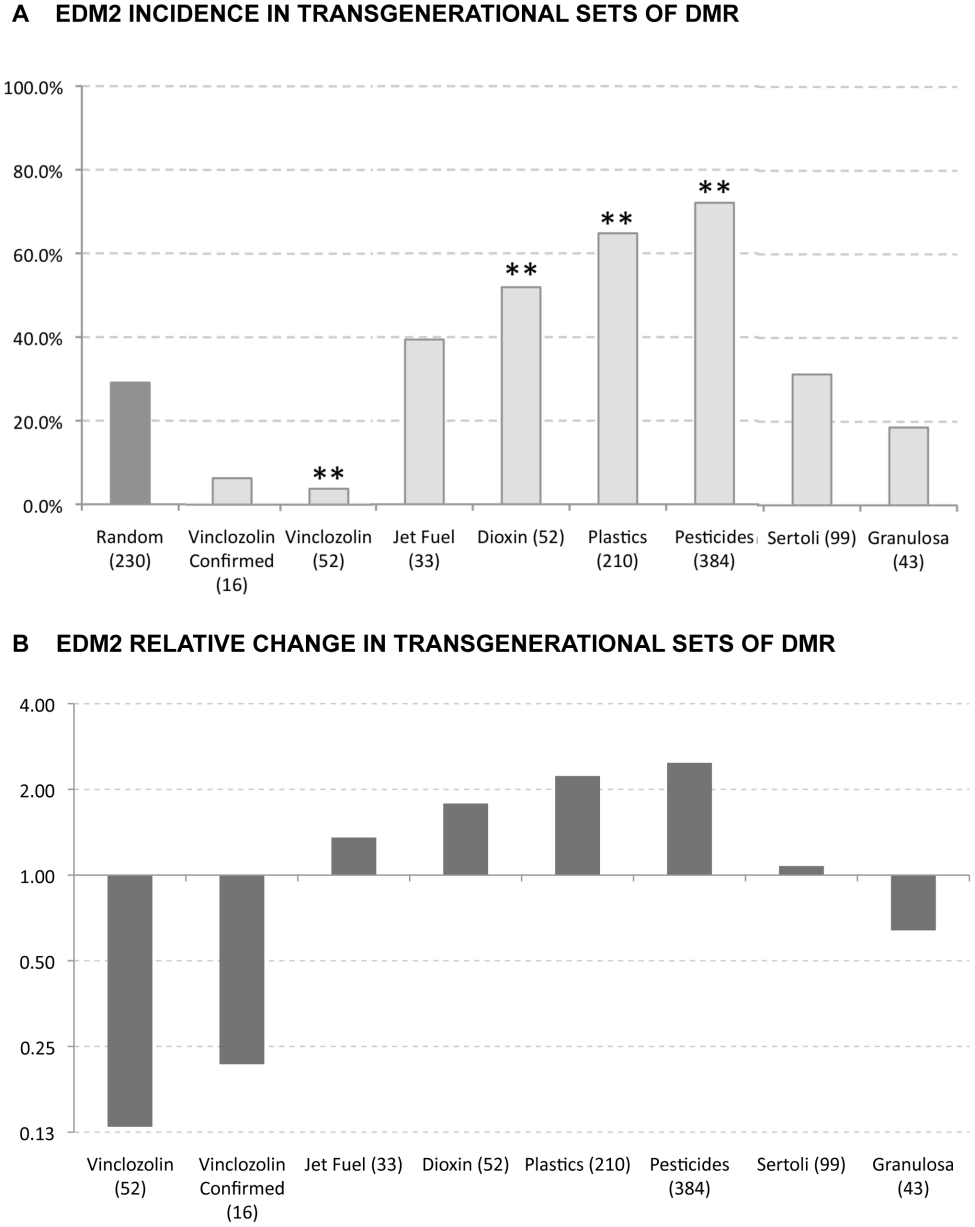
EDM2 incidence in exposure epimutation data sets. (A) Individual occurrence (percentage) of EDM2 in a variety of sets with transgenerational DMR and (B) relative change of EDM2 in these DMR. Columns with ** represent significant change with p<0.01, while columns with * represent significant change with p<0.05.

The presence of EDM1 was tested in a variety of transgenerationally altered DMR from sperm and from somatic Sertoli and granulosa cells [44], [45]. These transgenerational F3 generation DMR sets included vinclozolin (52 DMR) [3] dioxin (50 DMR) [2], hydrocarbons, jet fuel (33 DMR) [8], pesticide, permethrin and DEET (367 DMR) [5], plasticizers, BPA and phthalates (198 DMR) [46]. The somatic Sertoli cells and granulosa cells were obtained from F3 generation vinclozolin lineage animals. The DMR were identified with a comparative hybridization MeDIP-Chip analysis on F3 generation control versus exposures lineage cells. A subset of the vinclozolin F3 generation sperm DMR that were confirmed with bisulfite-mass spectrometry were also examined separately and termed “confirmed”. A computer generated random set of DNA sequences using the same genetic features of size and promoter association was created to act as a control for the comparisons (random occurrence). The presence of EDM1 was found to be significantly increased in the vinclozolin DMR set (52 sequences) and in the “confirmed” subset of 16 sequences [3] when compared to a random occurrence set of computer-generated sequences (Figure 2). Interestingly, EDM1 incidence tended to be decreased in sperm DMR from non-vinclozolin exposures or in sets from somatic cells when compared with its occurrence in a random set of sequences. Significant decreases are observed for the sperm plastics and pesticides DMR groups and for the somatic group of Sertoli cells from the vinclozolin exposure lineage (Figure 2A). The presence of EDM2 motif was also tested against the distinct sets of DMR sequences (Figure 3). It was found that EDM2 was significantly increased in the promoter associated sperm DMR sets of dioxin, plastics and pesticides lineages, and in vinclozolin lineage Sertoli cells when compared with its occurrence in a random set of sequences. The most significant increases were in the plastics and pesticides DMR groups with an over two-fold increase in EDM2 incidence. Therefore, two different motifs were identified with EDM1 being primarily associated with vinclozolin lineage DMR and EDM2 being predominant in a number of the other exposures.

**Figure 4:**
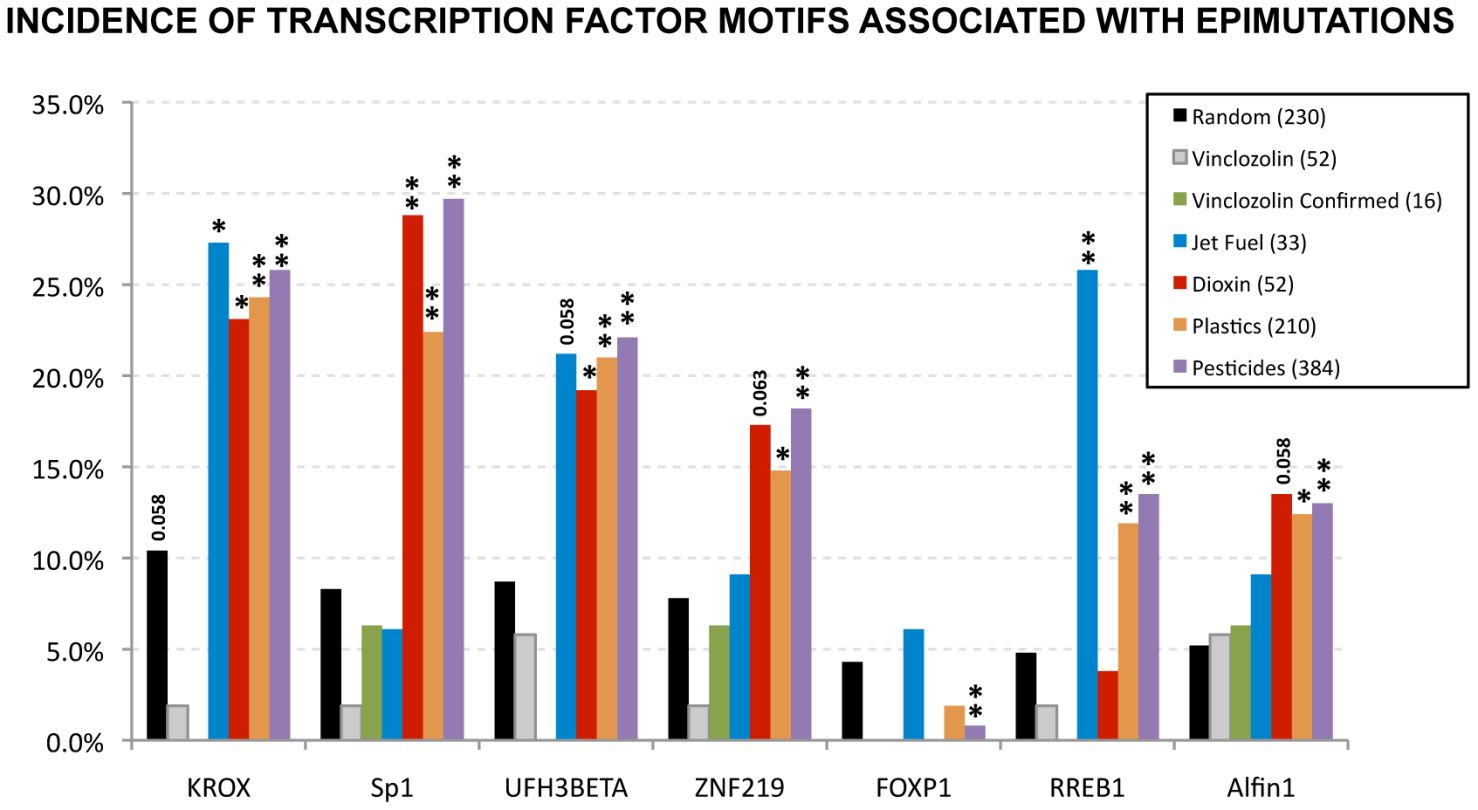
Incidence of consensus motifs of known transcription factor binding sites in DMR from a variety of F3 generation exposure lineage sperm DNA. Columns with (**) represent significant change with p<0.01, while columns with (*) represent significant change with p<0.05. For nearly significant changes p-values are shown in the respective column. Colored legend for specific exposure lineage DMR sets with total number indicated. The percentage incidence is indicated for the different transition factor sites.

The two DNA motifs identified from the analysis of the DMR groups were then compared to a database of eukaryotic transcription factor binding sites. The top five similarities of known transcription factors binding sites for each motif are shown in Table 1. The presence of motifs of these transcription factor binding sites was then tested against the different exposure lineage sets of DMR sequences (Figure 4). Observations indicate the KROX, SP1, UF1H3-beta and ZNF219 were consistently increased in dioxin, plastics and pesticides groups. However, their incidence in the jet fuel group was variable, with significant increases of only KROX in this group. RREB1 was observed in the jet fuel, plastics and pesticides groups. With the exception of UF1H3-beta, all these transcription factors are zinc fingers. FOXP1 was significantly decreased only in the pesticides group. Alfin1 was significantly increased in the plastics and pesticides group, and had a tendency to increase in the dioxin group. Therefore, zinc finger binding sites are apparently associated with the sperm transgenerational epimutations.

**Table 1:** Similarities of GLAM2 created motifs with known transcription factor for binding sites.

Sequence sets used to build the Glam2 Motif	Best Motif similarities in STAMP database with respective E-values				
	1st	2nd	3rd	4th	5th
**Vinclozolin**	AZF1	FOXP1	HMG-IY	STE11	BR-C
	1.44E-14	1.53E-10	5.09E-08	4.95E-07	2.11E-02
**Dioxin**	UF1H3BETA	KROX	RREB1	ZNF219	PAX
	3.41E-14	2.68E-08	2.01E-07	6.39E-07	49.56E-07
**Jet Fuel**	CDC5	KROX	Dde	IME1	HEN
	2.07E-05	4.26E-05	8.58E-05	9.53E-05	11.05E-04
**Pesticides**	ZNF219	PAX	KROX	UF1H3BETA	MAZ
	3.04E-10	41.50E-09	3.27E-08	1.74E-08	1.61E-07
**Plastics**	KROX	RREB1	PAX	ZNF219	UF1H3BETA
	1.25E-12	1.57E-11	41.39E-10	1.89E-09	8.94E-09

Similarity between motifs created from a variety of F3 generation exposure lineage sperm DNA and known transcription factor binding site matrices. The top five similarities are shown for each created motif with their respective statistically significant E-values.

**Figure 5:**
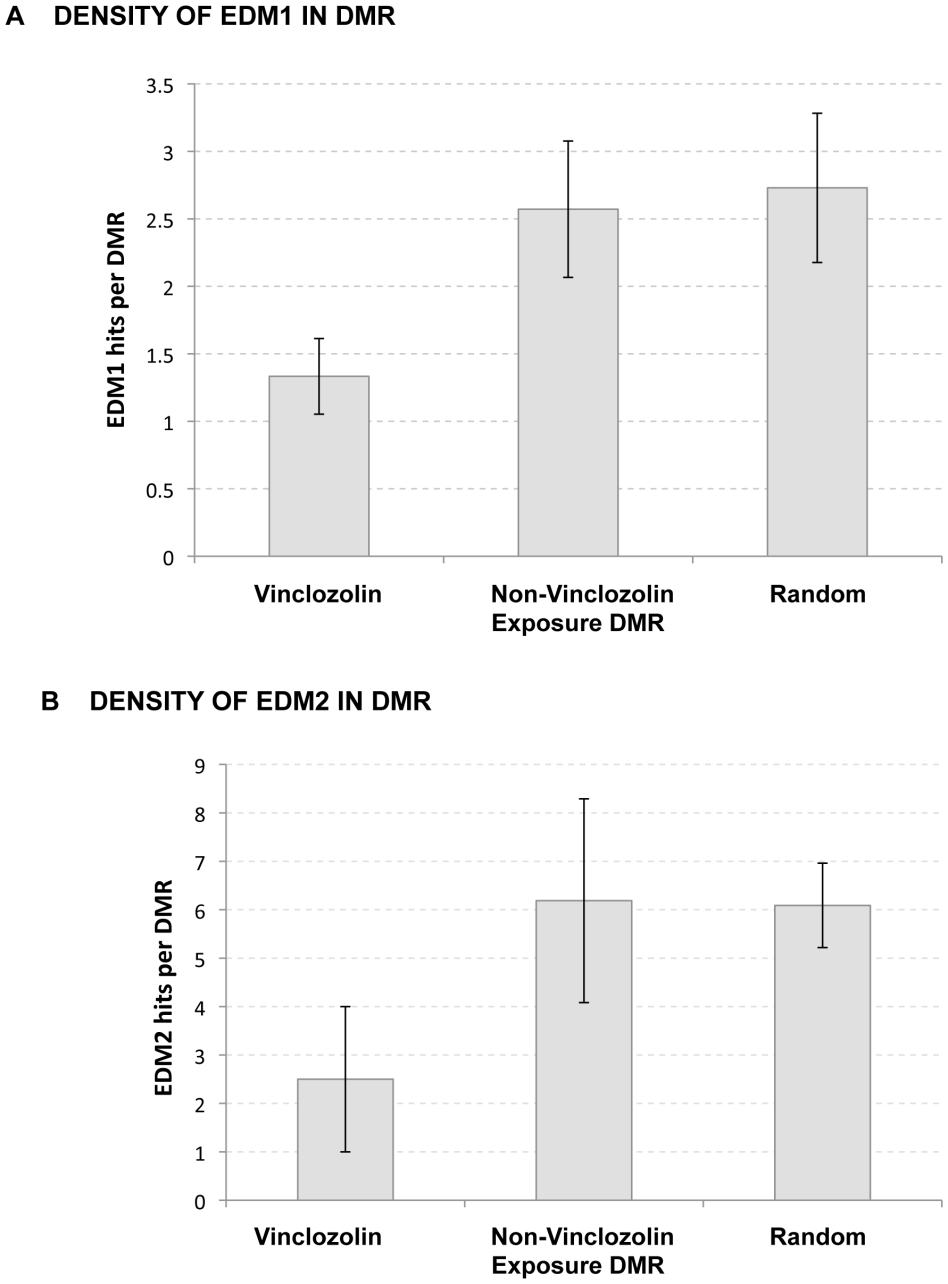
Density of incidences of (A) EDM1 and (B) EDM2 in sets of transgenerational DMR. The vinclozolin DMR, combination of plastics (BIP), pesticide (PIP), jet fuel (JIP) and dioxin (HIP) DMR, and a random set of genomic sites were investigated. The number of EDM1 or EDM2 sites per DMR is presented with the mean± SEM.

**Figure 6:**
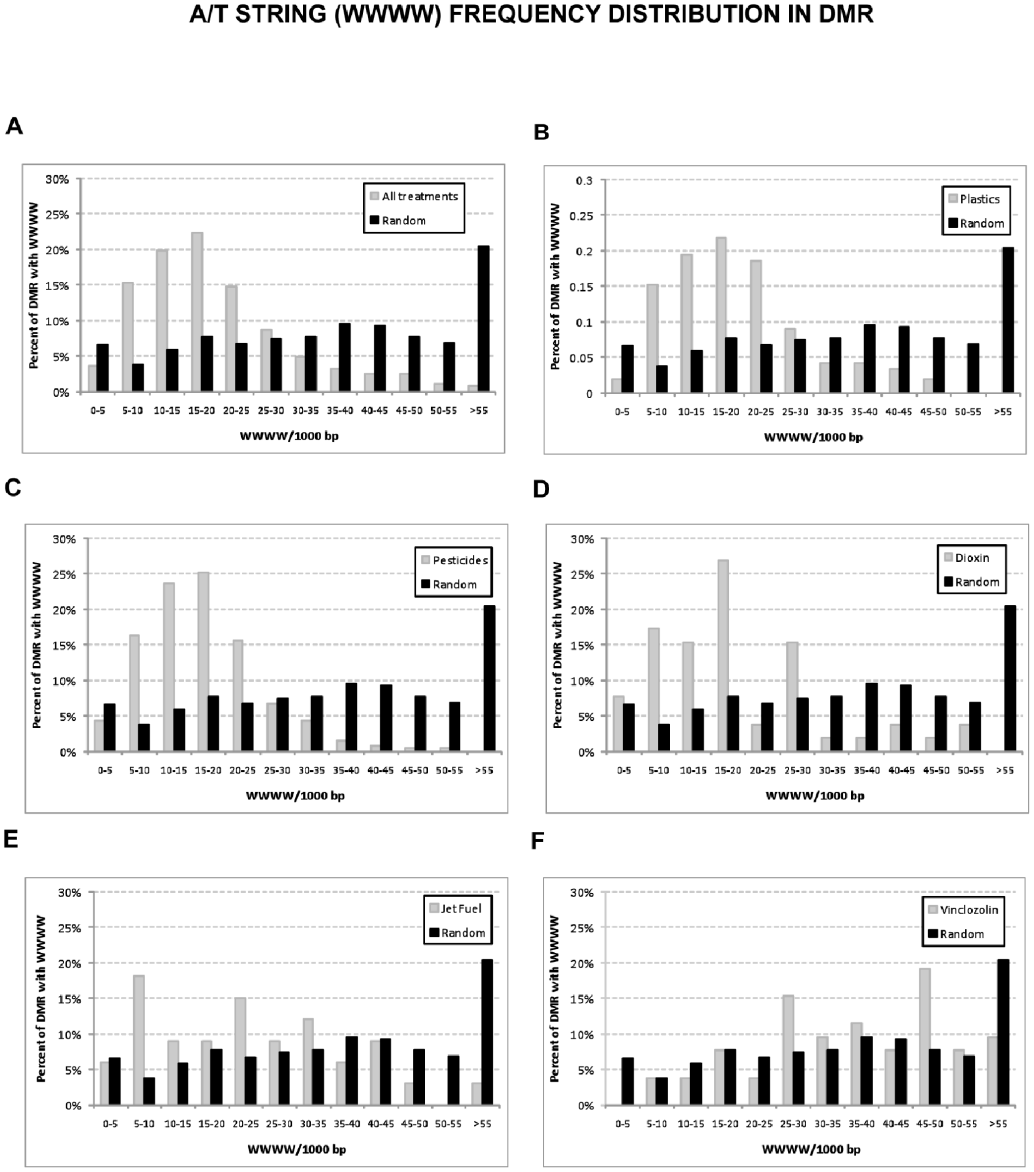
Distribution of A/T string (WWWW) incidence across the transgenerational DMR sets. The percent of DMR with A/T string sequences for all DMR (A), plastics (B), pesticides (C), dioxin (D), jet fuel (E), and vinclozolin (F) are presented compared to the random sequence data set.

The possibility that an altered density of EDM1 or EDM2 motifs could be observed in DMR sets versus the random set was examined. No significant changes were observed for the density of EDM1 or EDM2 in DMR from the exposure groups in reference to the random set (Figure 5). Given the composition of EDM1 in terms of being an A/T rich sequence and the reported role of A/T rich sequences as a recognition site for de novo DNA methylation [47], the density distribution of this feature across the different sets of DMR was also analyzed. Differences in the density distribution of A/T strings were found between the plastics, pesticides, dioxin and jet fuel groups and equivalent random sets of sequences. There is an overall reduction in the density of A/T strings in DMRs from these groups in comparison to the random set (Figure 6 A–E; p<0.01). However, A/T string density in the vinclozolin group is similar to the random distribution (Figure 6F).

**Figure 7:**
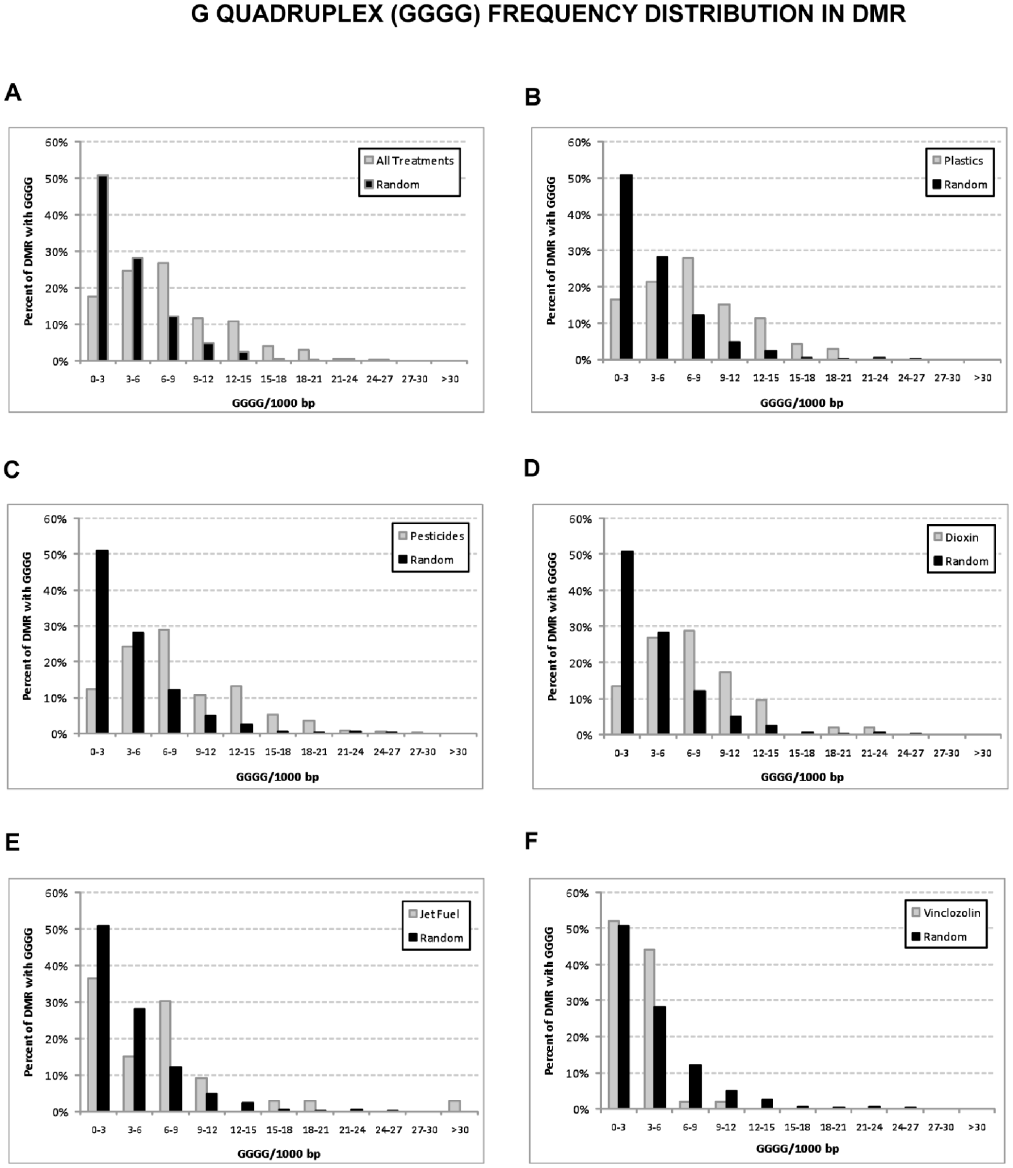
Distribution of G quadruplexes (GGGG) incidence across the transgenerational DMR sets. The percent of DMR with G-quadruplexes of sequences for all DMR (A), plastics (B), pesticides (C), dioxin (D), jet fuel (E), and vinclozolin (F) are presented compared to the random sequence data set.

EDM2 was observed to be a G/C rich sequence. Interestingly, previous reports show that G quadruplexes associate with zinc finger binding sites [48] and have a role in restricting DNA methylation [49] to influence chromatin dependent epigenetic instability [50]. Therefore, the distribution of G-quadruplexes across the different sets of DMR was analyzed. Interesting differences were also found in the distribution of G-quadruplexes in the exposure lineage DMR sets versus the random set of sequences. In the plastics, pesticides, jet fuel and dioxin groups an overall increase in G-quadruplex density regarding the random group was observed (Figure 7 A–E; p<0.01). The vinclozolin group had a distribution comparable with the random set (Figure 7F).

**Figure 8:**
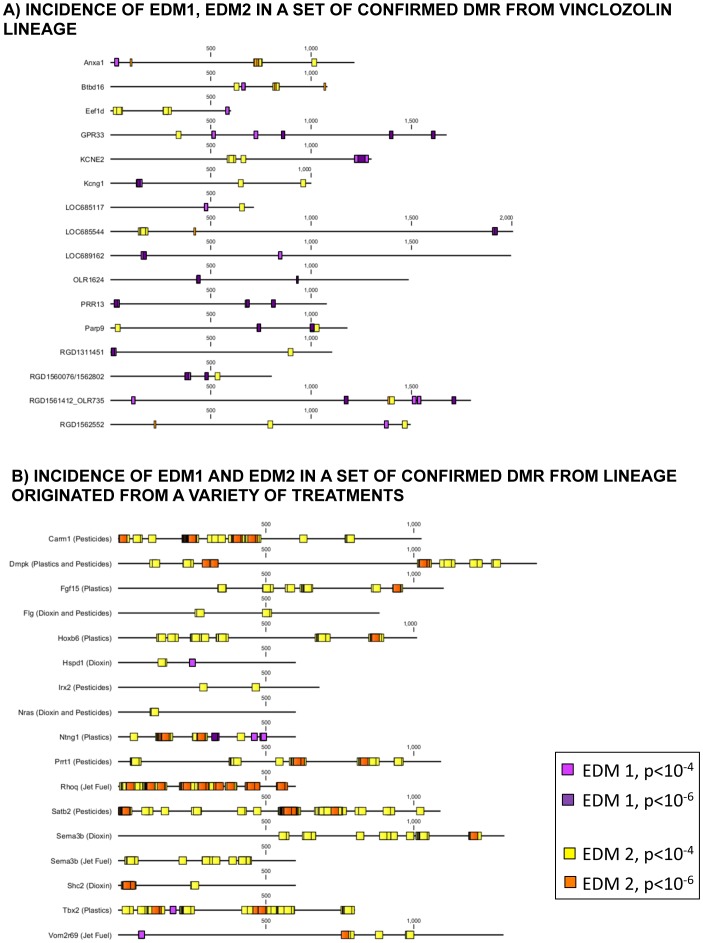
Visualization of DNA motifs associated with epigenetic transgenerational inheritance in selected sets of sequences: (A) incidence of EDM1 and EDM2 in a set of confirmed DMR from vinclozolin-lineage and (B) incidence of EDM1 and EDM2 in a set of confirmed DMR from lineage originated from a variety of exposures listed. The colored legend for EDM1 versus EDM2 motifs are presented.

**Figure 9:**
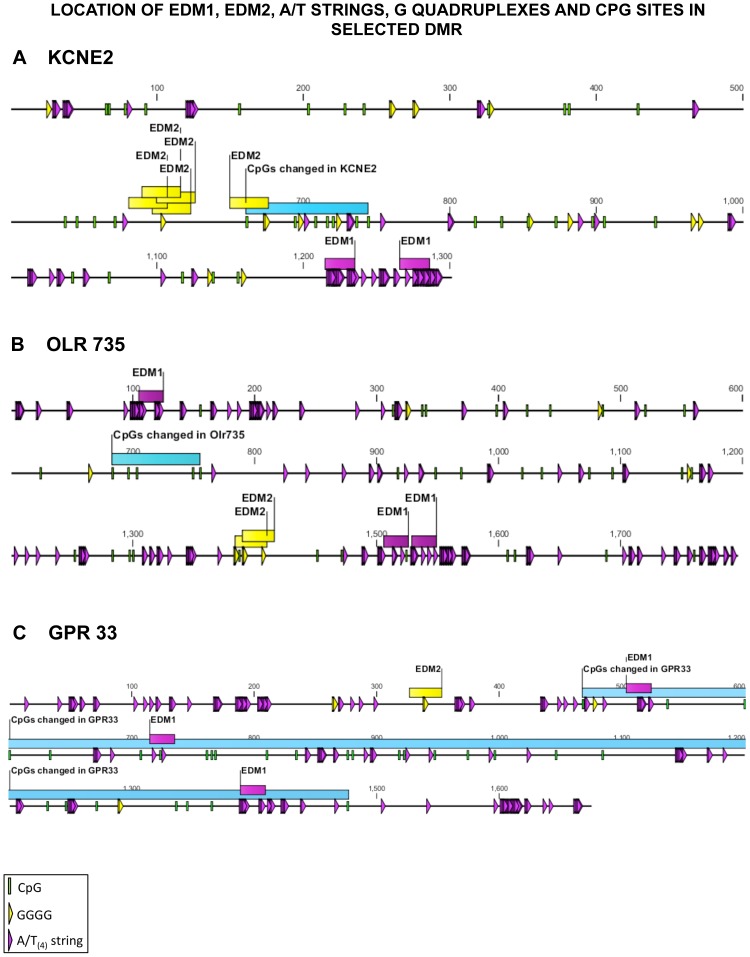
Location of EDM1, EDM2, G quadruplexes, A/T strings and CpG sites in selected DMR from the vinclozolin set: (A) KCNE2; (B) OLR 735; (C) GPR 33. The colored legend for CpG, GGGG sequence, and A/T string is presented, with the blue box being the DMR, yellow box EDM2, and purple box EDM1.

Schematic visualization of the features analyzed in selected sets of transgenerational DMRs previously confirmed for the vinclozolin and other exposure DMR groups are shown in Figure 8A,B. The locations of EDM1 and EDM2 in selected sequences are shown in Figure 8. Detailed schematic representations of the locations of these features in selected DMR from the vinclozolin lineage exposure are shown in Figure 9. Therefore, a number of genomic features were identified and appeared to be associated with the transgenerational sperm epimutations investigated.

A follow up experiment was done to help confirm the observations regarding the genomic features associated with the transgenerational DMR (i.e. epimutations). A more recently developed dichlorodiphenyltrichloroethane (DDT) induced transgenerational set of DMR in F3 generation sperm was investigated [51]. This DDT transgenerational DMR set was not used in the development of the EDM1 or EDM2 sequences, nor the other genomic feature identification. A comparison of the DDT DMR set with the random set of sequences demonstrated a 12.8% incidence in the presence of EDM1, which is similar to the random sequence incidence. A 64.1% incidence of EDM2 is observed, which represents a statistically significant increase (p<0.01) compared to the random sequence occurrence. The density distribution of the A/T strings decreased significantly (p<0.01) and G-quadruplexes increased significantly (p<0.01) in the DDT DMR compared to random sequences (Figure S1). Therefore, the patterns of incidence of EDM1, EDM2, G-quadruplexes and A/T strings in the DDT set are similar to the plastic, pesticide, jet fuel and dioxin DMR sets. Therefore, many of the same genomic features were also present in the transgenerational DDT sperm DMR [51] compared to the random sequences. Although further experiments are required to address the functional importance of these genomic features, this analysis helps confirm the presence of these genomic features in the environmentally induced epigenetic transgenerational inheritance of the sperm epimutations.

## Discussion

The phenomenon of environmentally induced epigenetic transgenerational inheritance is a germline mediated process [1], [2], [3], [4], [5], [6]. Germline epigenetic marks are altered during early fetal development and these environmentally induced epigenetic modifications (epimutations) can be transmitted to subsequent generations. Although the initial genomic feature associated with all the epimutations previously identified was a low CpG density (<12 GpG/100bp), other genomic features are anticipated. The current study is designed to perform a bioinformatic analysis and identify patterns of DNA sequences (motifs) in sperm and somatic cell DMR. Observations are anticipated to provide insights into the potential molecular mechanisms involved in establishing these epigenetic marks.

Identification of DNA sequence motif incidences was performed in different sets of previously identified sperm and somatic cell DMR sequences. Two motifs were identified that are associated with the DMR sets from different environmental exposures. Two different motifs were identified, EDM1, which is an A/T rich motif that is present in the transgenerational vinclozolin DMR set, and EDM2 that is a G/C rich motif present in the other germline transgenerational DMR sets investigated (jet fuel, pesticides, plastics and dioxin). The incidence of EDM1 is over-represented only in the vinclozolin lineage sperm DMRs, but is not over-represented in the somatic cell vinclozolin lineage DMR sets tested. These observations suggest the somatic cell epigenetic modifications are secondary to the germline epigenetic modifications and probably occur through alternate developmental mechanisms. EDM2 shows the opposite pattern of EDM1, being over-represented in the DMRs of all the exposure lineages, except for vinclozolin.

An analysis of known motifs with a database of transcription factor motifs shows that zinc finger motifs are associated with the sperm epimutations. Further analysis of the presence of these transcription factor binding sites in promoter DMR regions of the exposure sets shows that zinc finger transcriptome factor consensus DNA binding motifs for Krox, Sp1, Znf219 and Rreb1 are over-represented in the majority of the transgenerational sperm DMR sets investigated. These observations suggest that zinc finger containing DNA binding factors may have a role in the molecular mechanism of epigenetic transgenerational inheritance of sperm epimutations. UFH3Beta might also be relevant, since it follows the same patterns as these zinc finger binding factors. Interestingly, previous studies have highlighted the role of zinc fingers in epigenetic reprogramming. For example, the zinc-finger protein UHRF1 has recently been shown to have a role in maintaining DNA methylation in specific genomic regions in mammals [52]. Other studies have shown that the zinc-finger ZBTB4 preferentially bind to methylated DNA [53]. The potential that zinc finger binding regions may be targets for DNA methylation changes that will maintain DNA methylation alterations transgenerationally needs to be further investigated. DNA methylation changes in zinc finger rich regions may also preferentially occur in the germline. Another correlation of interest is FoxP1, which is reduced in all but one treatment (jet fuel). Interestingly, FoxP1 expression has been shown to be altered by the hypomethylating agent 5-azacytidine and by micro RNA expression neighboring the FoxP1 gene in human hepatocellular carcinoma cell lines [54]. The reduced incidence of FoxP1 sequences in the transgenerational DMR suggests the potential absence of epigenetic mechanisms that may correct the epigenetic defect induced. This may allow for these modifications in DNA methylation to be permanently transmitted to subsequent generations. These potential mechanisms need to be further investigated.

Another genomic feature analyzed was the density of the incidence of EDM1 or EDM2 within the DMR. Because of the possibility that even if over-representation of individual matches does not occur, a cluster incidence of these motifs might occur. The density of EDM1 or EDM2 in the DMR was determined and no difference was found between any of the exposure DMR groups and the random sequence set (Figure 5). Since EDM1 is A/T rich, the frequency of short A/T strings was also tested in the DMR sets. Interestingly, the incidence of A/T strings (WWWW) is less frequent than in the random sequence set for all the exposures but vinclozolin. In *Neurospora crassa* A/T-rich sequences are shown to be a fundamental recognition site for *de novo* DNA methylation [47]. A/T strings adjacent to CpGs seem to be a requirement for binding of some DNA binding proteins such as MeCP2 [55]. Therefore, the presence of the A/T string is a genomic feature that contributes to the susceptibility of the epimutations to develop and/or be transmitted.

The other DNA motif obtained was called EDM2 and it was found to have guanine rich regions. Previous studies have shown that CpGs with high methylation are generally not present in G-quadruplexes (GGGG), which suggests that DNA methylation is restricted when G-quadruplex features exist [49]. G-quadruplex unwinding is a conserved mechanism which prevents G-quadruplex-induced damages such as genetic and epigenetic changes [56]. Interestingly, the observations show that the incidence of G-quadruplexes is more frequent in the random sequence group than in all the exposure DMR groups, but vinclozolin. This G-quadruplex conformation forms pockets of accessibility that could open during specific times during development, allowing for epigenetic modifications to be established. Indeed the formation of G-quadruplex structures depends of the RAV1 factor, which when absent alters the incorporation of histones [50]. As mentioned above, zinc finger binding sites are enriched in the transgenerational sperm DMR. Interestingly, previous reports show that G-quadruplexes associate with zinc fingers [48]. Therefore, the presence of G-quadruplexes, zinc fingers and/or chromatin remodeling proteins appear to be associated with the transgenerational sperm epimutations.

The motif associated with the vinclozolin DMR (EDM1) was found to be distinct from the motif associated to the other exposure DMR (EDM2). One speculative mechanism to explain the difference is the variable signaling mechanisms of the compounds generating the transgenerational germline epimutations. While vinclozolin is a known anti-androgenic compound [57], several of the other compounds investigated are associated with estrogenic effects. The estrogenic effects of bisphenol A (BPA) and the phthalates have been established [58]. The action of both permethrin [59] and dioxin [60] are also reported to have estrogenic effects. Jet fuel (JP8) has been reported to reduce LH levels in women [61] by disruption of testosterone conversion to estradiol by aromatase [62]. Although the actions of the toxicants are on the F1 generation fetus, the altered epigenetic programming may be in part different due to the distinct signaling. This potential differential signaling effects on the germline epimutations needs to be further investigated.

An initial experiment to help validate the presence of these genomic features in the transgenerational sperm epimutations used a recent DDT sperm DMR set for analysis [51]. This DDT DMR set was not used in the identification of the genomic features. A number of the genomic features were also found to be present in the DDT sperm epimutations. The distribution of the genomic features in the DDT DMR group has a similar pattern to the plastics, pesticides, jet fuel and dioxin DMR groups. The mechanism of action of DDT is primarily to act as an estrogenic compound [63], which is generally similar to the actions of the other compounds, and distinct from the anti-androgenic actions of vinclozolin. This initial validation helps confirm the presence of the features, however, the functional role of these features remains to be elucidated.

Observations lead to the speculation that the mechanism of the transgenerational epigenetic programming of germline epimutations may be in part based on the action of zinc finger motifs and G-quadruplex sequences that can alter chromatin structure and accessibility to proteins. This alteration may allow an opening of DNA that alters the action of DNA methyltransferases or interacting factors. G-quadruplex regions would be more prone for this opening to epigenetic marks to occur. The zinc finger factors may interact with other proteins to promote this chromatin remodeling and/or altered expression of non-coding RNA. The current study identified a number of motifs and genomic features potentially associated with the DMR involved in the environmentally induced epigenetic transgenerational inheritance of sperm epimutations. Future studies are now needed to further investigate the specific proteins involved and developmental aspects of these epimutations.

## Methods

### DMR Sequence Sets

Exposure sets of DMR sequences used to perform the bioinformatic analyses were obtained from previous studies from our group showing transgenerational epigenetic changes in the F3 generation sperm and somatic cells [3], [6], [10], [45]. The DMR sequences used were from the published data sets, using a p-value cut-off of 10^−7^ instead of the p-value cut-off of 10^−5^ used in these publications. The DMR data sets were reduced in size, based on the above statistics, to allow for creation of the DNA motifs by the web-based tools used, which have size limitations. The GEO accession number (GSE57693) for these previous publications and additional information on the data access and bioinformatics can be found at www.skinner.wsu.edu/arrays.

### Motif Analyses

Two main computational methods exist to identify shared motifs in sets of sequences: (i) application of *ab initio* motif discovery algorithms, which search for recurring patterns in a set of DNA sequences and (ii) assessment of statistical over-representation of previously characterized motifs (from transcription factor binding site databases) in sequences [64]. In the present study the GLAM2 algorithm (Gapped Local Alignment of Motifs, available online on MEME suite) was used for *ab initio* motif discovery. GLAM2 considers insertions or deletions that are a variable and not incorporated by other algorithms [41]. The DMR sets were uploaded to the GLAM2 website and the best motif produced with the default settings was chosen for each sequence dataset. Previously, motifs were identified using GLAM2 in sets of sequences with vinclozolin-induced transgenerational changes in DNA methylation in the male germline [3], [4]. The web-based tool FIMO (Find Individual Motif Occurrences, available online on MEME suite) is a general-purpose web-based tool for identifying candidate binding sites [65]. FIMO assigns scores (p and q values) to individual motif occurrences in a defined set of sequences. FIMO was used in the present study to interrogate whether the motifs previously created with GLAM2 would be overrepresented in the tested sets of sequences versus a random set. FIMO was set with a p-value of 10^−6^ and the scan was on both strands. The sequences with matches of motifs were counted and the percentage of the sequences with matches was calculated in for each DMR dataset. The number of motif matches per sequence was also counted to determine the density of motif matches per DMR dataset. In addition to these analyses, comparison of created motifs with known motifs of transcription factors was performed with STAMP. STAMP is a web-based bioinformatic toolbox used to detect similarities of input motifs to motifs representing transcription factor binding sites, which are present in a database that include binding sites information from several organisms [43]. GLAM2 built motifs were compared to the ‘selected eukaryotic’ database of transcription factor motifs in STAMP, using the default settings. The top five matches (default setting) were selected (Table 1). These selected motifs were then scanned against the DMR sequence datasets using FIMO, as described above. Another feature of STAMP is that it groups motifs based on similarities using a familial binding analysis. This analysis was performed to contruct a tree of similarities between the GLAM2 built motifs (Figure 1). The incidence of G quadruplexes and A/T strings in DMR sets were computed with R (R Development CoreTeam (2010), R: A language for statistical computing, R Foun-dation for Statistical Computing, Vienna, Austria. ISBN 3-900051-07-0, URL http://www.R-project.org). For this, matches for GGGG and WWWW motifs were interrogated, respectively. The number of matches per sequences was then obtained and the density of matches per sequence sets was calculated. Figures showing visualization of all motif incidences (GLAM2 built, GGGG and WWWW) were created using CLC Workbench (Cambridge, MA).

### Statistical Analyses

Incidences of motifs between the DMR from different treatments and random sets were tested with Fisher’s test. Comparison of density of motif incidences between treatment sets of DMR and random sets were performed with Student’s t-test. The average of the distributions of G quadruplexes and A/T strings was also compared with Student’s t-test between the treatment DMRs and random sets.

## Supporting Information

Figure S1 Validation with DDT DMR data set. (A) Distribution of A/T string (WWWW) incidence in the DDT DMR data set. The percent of DMR with A/T string sequences are presented compared to the random sequence data set. (B) Distribution of G quadruplexes (GGGG) incidence in the DDT DMR data set. The percent of DMR with G-quadruplexes are presented compared to a random sequence data set.
(PDF)
