# Supplementary material for: Identification of Genomic Features in Environmentally Induced Epigenetic Transgenerational Inherited Sperm Epimutations
Source: PLoS One. 2014 Jun 17;9(6):e100194. doi: 10.1371/journal.pone.0100194 (PMC4061094; doi:10.1371/journal.pone.0100194)

Supplementary Figure S1

A **A/T String (WWWW) Frequency Distribution in DDT DMR**

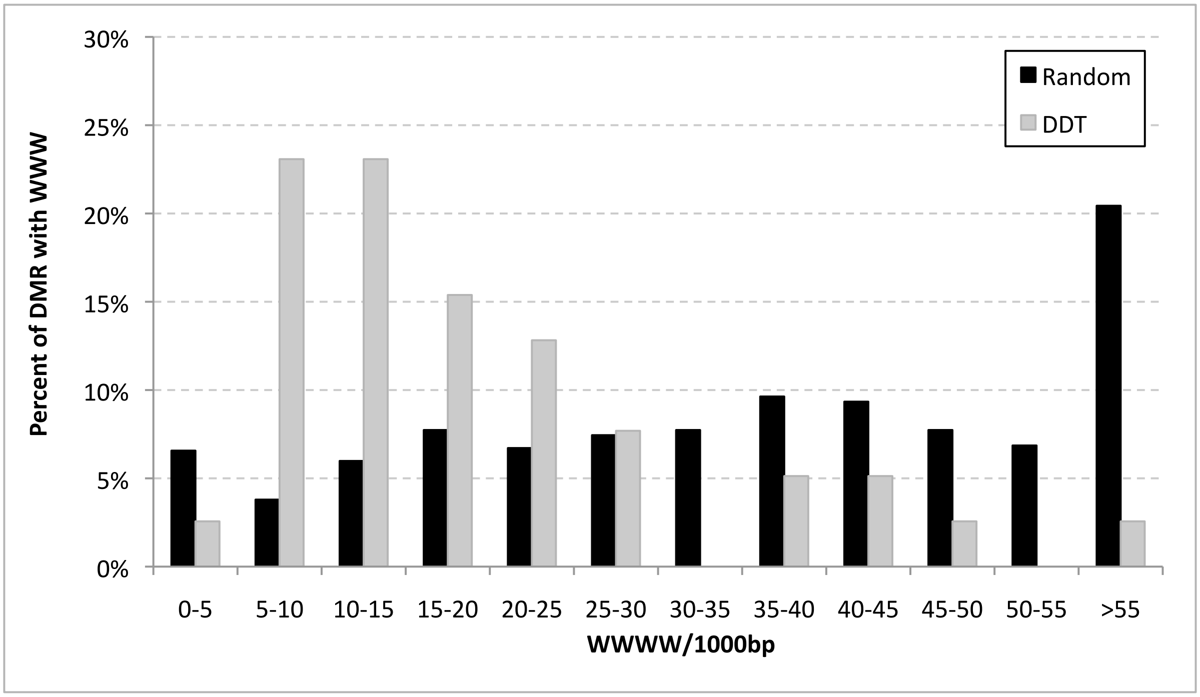

B **G-Quadruplex (GGGG) Frequency Distribution in DDT DMR**

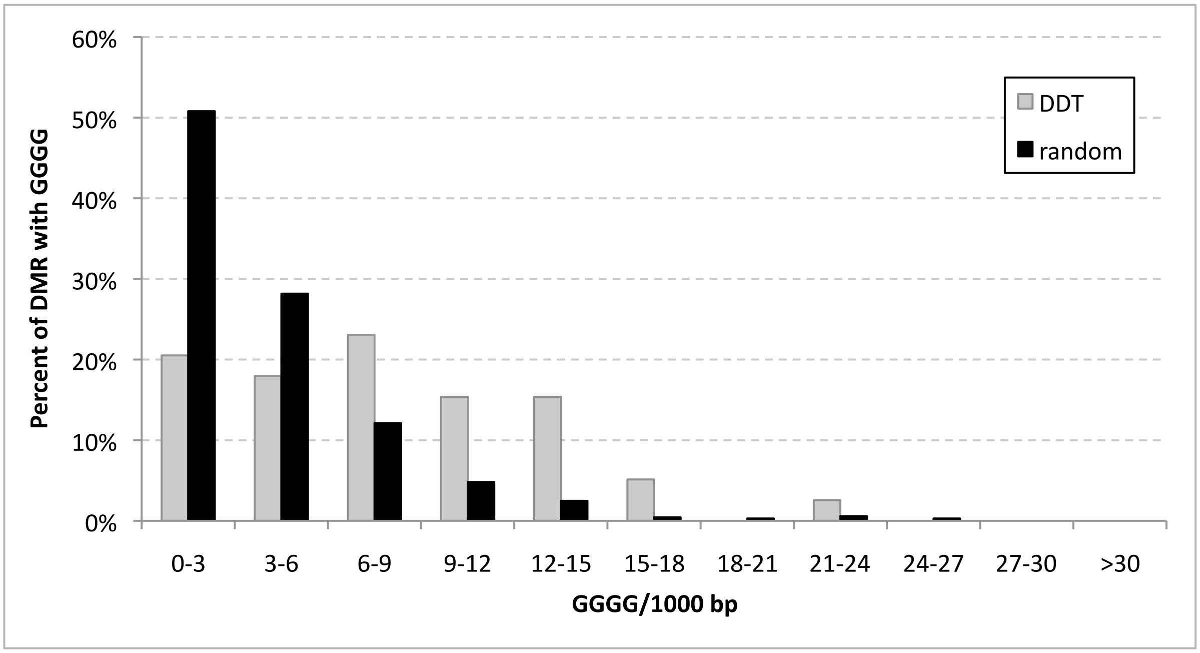

Supplement: Figure S1 — Validation with DDT DMR data set. (A) Distribution of A/T string (WWWW) incidence in the DDT DMR data set. The percent of DMR with A/T string sequences are presented compared to the random sequence data set. (B) Distribution of G quadruplexes (GGGG) incidence in the DDT DMR data set. The percent of DMR with G-quadruplexes are presented compared to a random sequence data set. (PDF) [file pone.0100194.s001.pdf]
